# The Possible Role of *Staphylococcus epidermidis* LPxTG Surface Protein SesC in Biofilm Formation

**DOI:** 10.1371/journal.pone.0146704

**Published:** 2016-01-22

**Authors:** Laleh Khodaparast, Ladan Khodaparast, Mohammad Shahrooei, Benoit Stijlemans, Rita Merckx, Pieter Baatsen, James P. O’Gara, Elaine Waters, Lieve Van Mellaert, Johan Van Eldere

**Affiliations:** 1 KU Leuven–University of Leuven, Department of Microbiology and Immunology, Laboratory of Clinical Bacteriology and Mycology, B-3000, Leuven, Belgium; 2 VUB—Vrije Universiteit Brussel, Laboratory of Cellular and Molecular Immunology B-1050, Brussels, Belgium; 3 VIB—Vlaams Instituut voor Biotechnologie, Department of Myeloid Cell Immunology, B-1050, Brussels, Belgium; 4 KU Leuven–University of Leuven, Center for Human Genetics, Electron Microscopy Facility, VIB Bio Imaging Core, B-3000, Leuven, Belgium; 5 National University of Ireland, School of Natural Sciences, Department of Microbiology, Galway, Ireland; 6 University of Liverpool, Institute of Infection and Global Health, Liverpool, United Kingdom; 7 KU Leuven–University of Leuven, Department of Microbiology and Immunology, Rega Institute of Medical Research, Laboratory of Molecular Bacteriology, B-3000, Leuven, Belgium; 8 KU Leuven–University of Leuven, University Hospitals Leuven, Department of Laboratory Medicine, B-3000, Leuven, Belgium; University of Iowa Carver College of Medicine, UNITED STATES

## Abstract

*Staphylococcus epidermidis* is the most common cause of device-associated infections. It has been shown that active and passive immunization in an animal model against protein SesC significantly reduces *S*. *epidermidis* biofilm-associated infections. In order to elucidate its role, knock-out of *sesC* or isolation of *S*. *epidermidis sesC*-negative mutants were attempted, however, without success. As an alternative strategy, *sesC* was introduced into *Staphylococcus aureus* 8325–4 and its isogenic *icaADBC* and *srtA* mutants, into the clinical methicillin-sensitive *S*. *aureus* isolate MSSA4 and the MRSA *S*. *aureus* isolate BH1CC, which all lack *sesC*. Transformation of these strains with *sesC* i) changed the biofilm phenotype of strains 8325–4 and MSSA4 from PIA-dependent to proteinaceous even though PIA synthesis was not affected, ii) converted the non-biofilm-forming strain 8325–4 *ica*::*tet* to a proteinaceous biofilm-forming strain, iii) impaired PIA-dependent biofilm formation by 8325–4 *srtA*::*tet*, iv) had no impact on protein-mediated biofilm formation of BH1CC and v) increased *in vivo* catheter and organ colonization by strain 8325–4. Furthermore, treatment with anti-SesC antibodies significantly reduced *in vitro* biofilm formation and *in vivo* colonization by these transformants expressing *sesC*. These findings strongly suggest that SesC is involved in *S*. *epidermidis* attachment to and subsequent biofilm formation on a substrate.

## Introduction

Of all coagulase-negative staphylococci, *Staphylococcus epidermidis* is the most common cause of infections associated with catheters and other indwelling medical devices [1, 2]. It is a permanent and ubiquitous colonizer of human skin, can easily contaminate the medical devices during insertion, and subsequently form a biofilm [2, 3]. The capacity to form a biofilm is considered as one of the major virulence factors of this bacterial species [4, 5].

Staphylococcal biofilms develop via a multifactorial process, which may differ between species and strains. Nevertheless, most of the factors involved are analogous in *S*. *epidermidis* and *S*. *aureus* and have a similar function in biofilm formation [1, 2, 3].

Up to now, based on extracellular matrix macromolecules constituting the biofilm, three mechanisms of biofilm formation in staphylococci are identified [6]. Production of polysaccharide intercellular adhesin [PIA, also called poly-N-acetylglucosamine (PNAG)] was the first and for a long time, the only mechanism of biofilm formation identified [7, 8]. Further studies showed the existence of other PIA- or *ica*-independent mechanisms in *S*. *aureus* and *S*. *epidermidis*. Based on *in vivo* and *in vitro* studies, the proteinaceous biofilm formation was identified. In this case, the cell-surface and cell-cell attachment is based on proteins [9, 10]. More recently, a third mechanism based on extracellular DNA (eDNA) constituting a cell-to-cell or cell-to-substratum connecting component was recognized. This eDNA originates from autolysis [11, 12].

It has been shown that staphylococcal surface proteins such as accumulation-associated protein (Aap), biofilm-associated proteins (Bap, and Bap homologue Bhp), extracellular matrix-binding protein (Embp), fibronectin- or fibrinogen-binding proteins (FnBPA, FnBPB and Fbe/SdrG), and the major autolysin (AtlE) mediate the formation of the network of multilayered cell clusters and filamentous proteins, and thus play an important role in the biofilm accumulation phase [7, 10, 13, 14]. In *S*. *epidermidis* and *S*. *aureus*, LPxTG motif-containing proteins which covalently link to the cell wall via sortase activity, are determinants in the pathogenesis of device-related infections [7].

Through unknown or not well-characterized mechanisms such as insertion and excision of the insertion sequence IS*256* at specific hot spots of the *S*. *epidermidis icaA* and *icaC* genes, PIA/PNAG production, biofilm formation and biofilm phenotype may be phase variable, allowing to switch from PIA-dependent to proteinaceous phenotype [10, 15, 16]. In 2001, Knobloch *et al*. reported that NaCl affects biofilm formation through the activation of the σ^B^ operon, an important regulator of the *ica* operon, and thus can be used to distinguish *ica*-dependent from *ica*-independent biofilm formation [9, 17, 18]. By using dispersing agents such as sodium metaperiodate (SM), proteinase K (PK) and DNase I, the chemical composition of the biofilm extracellular polymeric substance can be determined and one can discriminate between PIA-dependent, proteinaceous and eDNA-based biofilms [9, 19].

So far, the roles of 5 *S*. *epidermidis* LPxTG proteins (Aap, Bhp, SdrF, SdrG, SesI) in the pathogenesis of *S*. *epidermidis* infections and biofilm formation have been studied [20, 21, 22]. We focused our research on the LPxTG motif-containing *S*. *e**pidermidis* surface protein SesC, a 676-amino acid (aa) protein with a predicted molecular mass of 75 kDa. The cytoplasmic precursor of SesC contains a 35-aa N-terminal signal peptide, required for Sec-dependent secretion and is cleaved off by the signal peptidase. The 37-aa C-terminal LPxTG-sorting signal is recognized by sortase, which will cleave the bond between the Thr and Gly and thereafter covalently link the 608-aa (68 kDa) remaining protein to the peptidoglycan layer. Using antibodies against the mature domain of the SesC protein, we were able to reduce *S*. *epidermidis* biofilm formation *in vitro* [23]. In addition, active and passive immunization against SesC could significantly reduce their biofilm formation on catheter fragments in animal models of subcutaneous and intravascular catheter infection [23]. However, the involvement and exact function of SesC in *S*. *epidermidis* biofilm formation have remained unknown, so far. In order to elucidate its role, knock-out of *sesC* or isolation of *S*. *epidermidis sesC*-negative mutants were attempted, however without success. Therefore, as an alternative strategy *sesC* was introduced into *S*. *aureus* strains and the effect of *sesC* expression in biofilm formation by these host strains was studied.

## Materials and Methods

### Bacterial strains, plasmids and media

Cloning experiments were performed in *Escherichia coli* DH5α competent cells (Invitrogen). *E*. *coli* DH5α transformants were grown in Lysogeny Broth (LB) or on LB agar at 37°C supplemented with ampicillin (100 μg/ml), as all plasmids used in this study (Table 1) contain an ampicillin resistance (*bla*) gene. All *Staphylococcus* strains (Table 1) were grown in brain heart infusion (BHI) medium or agar, and for biofilm formation assays also in BHI medium supplemented with 4% NaCl (BHI-NaCl) or 1% glucose (BHI-glucose). Bacterial CFU counting was done on Tryptone Soya agar (TSA, Oxoid) or blood agar plates (BD Biosciences). Whenever required, growth media were supplemented with appropriate antibiotics as follows: chloramphenicol at 10 μg/ml, erythromycin at 10 μg/ml and tetracycline at 5 μg/ml. Species identification and antibiograms for all clinical isolates were performed using a VITEK^®^ 2 automated system (bioMérieux).

**Table 1:** *Staphylococcus* strains and plasmids used in this study.

Strains	Characteristic(s)	Reference
***- S*. *epidermidis***		
10b	Clinical isolate	[24]
***- S*. *aureus***		
RN4220	Restriction-negative derivative of 8325–4	[25]
8325–4	NCTC8325 cured of prophages. 11-bp deletion in *rsbU*.	[9]
BH1CC	MRSA clinical isolate. Biofilm positive. SCCmec type II, MLST type 8, clonal complex 8.	[9]
MSSA4	Clinical isolate	This study
8325–4 *ica*::*tet*	*icaADBC*::*tet*; isogenic mutant of 8325–4	[9]
8325–4 *srtA*::*tet*	*srtA*::*tet*; isogenic mutant of 8325–4	[26]
BH1CC *ica*::*tet*	*icaADBC*::*tet*; isogenic mutant of BH1CC	[9]
BH1CC *srtA*::*tet*	*srtA*::*tet*; isogenic mutant of BH1CC	[26]
**Plasmids**		
pCN68	*E*. *coli*-*Staphylococcus* shuttle vector	[27]
pSRsrtA5	*E*. *coli*-*Staphylococcus* shuttle vector	[26]

### Cloning and expression of *S*. *epidermidis sesC* and *sesK* genes in *S*. *aureus* strains

The coding regions of *S*. *epidermidis sesC* (SE2232, Gene ID 1056520) and *sesK* (SE1501, Gene ID 1056680), were amplified using *sesC-* and *sesK-*specific primers (Table 2) containing additionally a *Sal*I or *Sma*I restriction site for cloning purposes. Genomic DNA (gDNA) of biofilm-forming *S*. *epidermidis* strain 10b, a clinical isolate [24], was used as a template. The amplicons were ligated into *Sal*I/*Sma*I-digested pCN68 *E*. *coli*—*Staphylococcus* shuttle vectors [27] yielding pCN*sesC* and pCN*sesK*. In this plasmid, P*bla*Z is the promoter. It is a highly active constitutive promoter; erythromycin was used as the selection marker. All recombinant plasmids were replicated in *E*. *coli* DH5α. Correctness of cloning was confirmed by restriction enzyme digestion, PCR, and nucleotide sequence analysis of the insert. Plasmids harvested from *E*. *coli* were first electroporated into the restriction-deficient *S*. *aureus* strain RN4220 and subsequently into other *S*. *aureus* strains. Presence and expression of *sesC*, *sesK*, *sasF* (Gene ID: 5775591), and *icaA* (Gene ID: 5776135) in transformed strains were evaluated using gel-based reverse transcription-PCR (RT-PCR) and Western blotting assays. Plasmid, gDNA and RNA isolation from bacterial strains and cDNA synthesis were performed as previously described [28].

**Table 2:** Primers used in this study

Name	Sequence (5’ → 3’)	RE site
**sesCFs sesCF**	AT*GTCGAC*TTTATTAAAGGAGT**ATG**TGTAAATG	*Sal*I
**sesCR**	AT*CCCGGG*TGATGATGC*CTA*TTACTATATATAA	*Sma*I
**sesKF**	AT*GTCGAC*GACCTCTTAACTAATT**ATG**TTATG	*Sal*I
**sesKR**	AT*CCCGGG*TCTCGTTATTTTCACTCAAATATC	*Sma*I

(Underlined sequences: restriction sites; bold sequence: start codon)

### Biofilm formation assay

The amount of biofilm formed by the different strains was determined using a semi-quantitative adherence assay in 96-well polystyrene microtiter plates (BD Biosciences) as previously described [23, 26, 28]. Briefly, 20 μl of stock cultures were inoculated into 5 ml (selective) BHI medium and grown to the end-exponential growth phase in a shaking incubator at 37°C. Cultures were subsequently diluted to an OD_600_ of 0.005 (5.x10^6^ CFU/ml) in fresh BHI medium whether or not supplemented with 4% NaCl or 1% glucose. 200 μl of the diluted cultures of bacteria were pipetted into sterile 96-well polystyrene microtiter plates and incubated overnight at 37°C without shaking.

After incubation, the wells were rinsed 3 times with phosphate-buffered saline (PBS) and dried afterwards. The adhered material was stained with 200 μl of a 1% (w/v) crystal violet (Sigma) solution for 10 min, and subsequently, the wells were washed 3 times with water and again dried. For quantification, 160 μl of 30% (v/v) acetic acid solution was added to each well to dissolve the crystal violet. The OD_595_ of the dissolved stain was measured in a multipurpose UV/VIS plate reader (VICTOR3 _TM_; PerkinElmer).

### Biofilm treatment assays

The biofilm stability against sodium metaperiodate (SM), proteinase K (PK) or DNase I treatment was tested as described previously [29, 30, 31]. Briefly, 200 μl of an overnight grown culture diluted to an OD_600_ of 0.005 in BHI-glucose, were pipetted into sterile 96-well polystyrene microtiter plates and statically incubated overnight at 37°C. After 24 h incubation, the growth medium was replaced with 200 μl solution of SM (10 mM in 50 mM sodium acetate), of PK (Qiagen GmbH, 1 mg/ml in 100 mM NaCl, 20 mM Tris/HCl, pH 7.5) or of DNase I (Sigma, 2 mg/ml in 5 mM MgCl_2_) Subsequently, plates were incubated at 37°C for 2 h and the remaining biofilms were quantified as explained above.

To assess the effect of specific anti-SesC antibodies (αSesC-IgGs) produced as earlier described [23] on biofilm formation, 1x10^6^ bacteria were in the first instance incubated with αSesC-IgGs (20 μg/ml bacterial suspension) for 2 h at 4°, and in a volume of 200 μl medium brought into a 96-well plate. Plates were incubated overnight at 37°C without shaking to allow bacterial growth and biofilm formation.

### PIA quantification by PIA non-specific immunoblot assay

The relative amount of PIA present in a biofilm was determined as described [31], however with some modifications. Briefly, 1 ml of a diluted overnight culture of bacterial suspension (5x10^6^ CFU/ml) in BHI-NaCl or BHI-glucose were pipetted in 24-well polystyrene microtiter plates (BD Biosciences) and next, plates were incubated overnight at 37°C. After incubation, spent medium was removed, 500 μl PBS was added into each well and the biofilm mass was removed from the surface via pipetting. Samples were transferred to 1.5 ml tubes which were next centrifuged for 3 min at 12000×*g*. Pellets and biofilm material were re-suspended in 0.5 M EDTA (pH 8) to an OD_600_ of 0.5 and PIA was extracted by boiling the samples for 5 min. After centrifugation at 18000×*g*, 250 μl of the supernatant was added to an Eppendorf tube with 25 μl PK solution (20 mg/ml). The mixture was incubated for 1 h at 60°C and afterwards PK was deactivated for 30 min at 80°C. Sample aliquots were applied to a nitrocellulose membrane, which was blocked with 5% (w/v) bovine serum albumin (BSA) in TTBS [Tris-buffered saline (100 mM Tris/HCl, 0.9% NaCl) with 0.05% Tween 20]. After washing the membrane 3 times in TTBS, it was incubated overnight at 4°C with wheat germ agglutinin-horseradish peroxidase conjugate (EY laboratories) in 1% (w/v) BSA-TTBS. After washing the membrane 3 times in TTBS, the presence of PIA was detected by the addition of Western blotting detection reagent (Amersham^TM^ ECL, GE Healthcare), and visualized with a ChemiDoc™ XRS+ System (Bio-Rad).

### Detection of SesC

Western blot analysis of SesC was performed as described [32]. Cells of 10 ml overnight bacterial cultures grown at 37°C in BHI-glucose were harvested by centrifugation, resuspended within lysis buffer [10 mM Tris/HCl, 10 mM EDTA, 1 mM phenylmethanesulfonyl fluoride (PMSF), pH 7.5, 100 μg lysozyme/ml, 100 μg lysostaphin/ml] and incubated for 1 h at 37°C. Then, cells were broken by passing them three times through a French press at 69 MPa (SLM Aminco) followed by sonication (Branson 2510, 42 kHz) of samples on ice. Proteins were electrophoretically separated on a 12% sodium dodecyl sulphate polyacrylamide gel and subsequently transferred onto a PVDF membrane. The primary antibody (1:5000 dilution of rabbit anti-SesC polyclonal antibodies in TTBS) was added to the membranes, which were left overnight, followed by horseradish peroxidase-conjugated anti-rabbit IgGs as secondary antibodies [1:2500 dilution in TTBS+1% (w/v) skim milk] for 2 h. Finally, the presence of SesC was visualized using ECL Western blotting detection kit (GE Healthcare) in combination with a ChemiDoc™ XRS+ System (Bio-Rad).

### Scanning electron microscopy

*In vitro* biofilm formation on cover Glasses (Ø 10 mm, Menzel GmbH) was visualized by scanning electron microscopy (SEM) as described [33]. Briefly, an overnight bacterial culture was diluted in BHI-glucose to an OD_600_ of 0.005, and 1 ml of the diluted culture was pipetted into the wells of sterile 24-well polystyrene microtiter plates, which each contained a glass disk. Plates were incubated overnight at 37°C without shaking, after which the disks were washed 3 times with PBS. Biofilms formed on the disks were fixed with 2.5% glutaraldehyde in 0.1 M sodium cacodylate buffer (pH 7.4) by incubation for 2 h at room temperature. After fixation, disks were rinsed with 0.1 M sodium cacodylate buffer (pH 7.4) for 30 min with three changes. Thereafter, a post-fixation step was done with 1% osmiumtetroxide for 2 h at 4°C. Next, disks were rinsed with distilled water (2 times, 10 min) and then dehydrated in 10 min steps in a series of ascending ethanol baths (25%, 50%, 75%, 95% and 100%). Following a bath of hexamethyldisilazan, dehydrated air-dried samples were mounted on support stubs with C-stickers and silver glue, and sputter coated with platinum (Agar Scientific, Auto Sputter Coater). Finally, the samples were observed and images taken with a JSM7401F field emission scanning electron microscope (JEOL) in a high vacuum mode with a conventional Everhart-Thornley detector at 5kV accelerating voltage.

### Investigation of the effect of SesC on the *in vitro* attachment to a catheter surface

*In vitro* bacterial attachment to the surface of a commercial polyurethane (PU) intravenous catheter (Arrow International) was examined as described [23, 34] with some modifications. Overnight cultures of *S*. *aureus* strains 8325–4 and 8325–4 (pCN*sesC*) were washed with saline (0.9 % NaCl) and diluted to an OD_600_ of 0.03 in saline. Seven mm catheter fragments were added to 2ml of bacterial suspension and the mixture was incubated at 37°C. After 2 h incubation, catheters were removed. After gentle rinsing with saline, catheters were placed in a tube containing 1 ml saline. Tubes were vortexed for 10 s, sonicated for 10 min at 40 kHz using a Branson water bath and again vortexed for 10 s. Thereafter, tube contents were 10-fold serially diluted and 50 μl aliquots of each dilution were plated on TSA plates using a spiral plater system (Spiral Plater Systems, Inc. Cincinnati, Ohio), and plates were incubated at 37°C overnight. Colonies were counted and the number of bacteria was defined as the mean of at least five quantitative cultures.

### Jugular vein catheterized (JVC) mouse model

In order to investigate the involvement of SesC in catheter-related infections (CRIs) *in vivo*, we used a central venous catheter murine model [28, 35, 36] that reflects the clinical situation of catheter colonization by contaminated infusions. Briefly, 4-weeks-old Swiss-Webster mice (Taconic) were anesthetized with a single intraperitoneal (i.p.) injection of sodium pentobarbital (Nembutal, 40–60 mg/kg body weight) and placed on a heating pad to maintain the body temperature at 37°C. An anesthetized and surgically prepared animal was then placed in the dorsal recumbence under a dissecting microscope (Zeiss, Jena, Germany, 10x magnification). A small vertical incision was made using small scissors and the right jugular vein was identified, mobilized and exposed with blunt surgical dissection. A single lumen Intramedic polyethylene catheter (Becton Dickinson #427400; internal Ξ 0.28 mm, outer Ξ 0.61 mm, insertion length, 1 cm) was inserted into the right jugular vein via a small incision in the vein made with vein scissors and advanced into the superior vena cava. A ligature was then tied loosely around the catheter and the patency was verified. Once blood flow had been established, the catheter was anchored in place. Subsequently, a small midline skin incision was made between the scapulae. The catheter was subcutaneously tunneled by a straight surgical clamp and exteriorized through midline scapular incision. The incisions were then closed with stitches. The patency was tested and the catheter was flushed with 100 μl of saline, sealed with a plug and left in place throughout the experiment. Thereafter, mice were housed separately and monitored for recovery. 24h after surgery, the mice were inoculated via the catheter lumen with 100 μl of an *S*. *aureus* suspension (OD_600_ of 0.03 (3.10^7^CFU/ml)). Then the catheter was flushed again with 100 μl saline so that bacteria entered the venous system of animals. At least every 12 h, animals were monitored for adverse events and all efforts were made to minimize animal suffering. All surgical procedures were performed under anesthesia with sodium pentobarbital (Nembutal) diluted in saline. After taking the blood samples at day 1 or 5 post-infection, animals were euthanized by CO_2_ inhalation and catheters and organs (spleen, liver, heart, vein and right kidney) were aseptically harvested from the animals. The organs were mechanically homogenized in saline and the portion of the catheter inserted into the vein (about 1 cm) was cut, gently washed and placed in a tube containing 1 ml saline. Tubes containing the catheter fragments were vortexed for 10 s, sonicated for 5 min at 40 kHz and again vortexed for 10 s. Serial dilutions of the organ homogenates and catheter fluid collections were cultured on blood agar plates using the spiral plating system, and plates were incubated at 37°C overnight. Colonies on all plates were counted and the number of bacteria was defined as the mean of at least 5 quantitative cultures. All *in vivo* experiments were repeated at least twice and conducted in compliance with the guidelines for animal experimentation. The Institutional Animal Care Commission and Ethical Committee of the KU Leuven approved all experimental protocols.

#### Study of the involvement of SesC in virulence and biofilm formation

Nine mice were divided into 3 groups of 3 mice. Overnight culture cells of *S*. *aureus* strains 8325–4, 8325–4 (pCN) and 8325–4 (pCN*sesC*) grown to the late exponential/early stationary growth phase in (selective) BHI medium and the cells were pelleted, re-suspended and diluted to an OD_600_ of 0.03 (circa 3x10^7^ CFU/ml) in 0.9% NaCl with the suitable antibiotics. After the 24-h recovery period, animals in one group were inoculated through the catheter lumen with 100 μl of one of the 3 prepared bacterial suspensions. Five days post-infection, the infection rate and the bacterial load on the implanted catheter were determined and compared between the 3 groups.

#### The effect of rabbit polyclonal αSesC-IgGs on infection rates and biofilm formation

Bacterial suspensions of *S*. *aureus* strains 8325–4 and 8325–4 (pCN*sesC*) were freshly prepared and diluted till an OD_600_ ~ 0.03. The diluted suspensions were incubated for 2 h at 4°C without IgGs, with αSesC-IgGs (80 μg/ml) or pre-immune IgGs (80 μg/ml). Fifteen mice divided into 5 groups of 3 mice groups received 100 μl of inoculum: i) 8325–4 without any IgG; ii) 8325–4 pre-incubated with αSesC-IgGs (80μg/ml); iii) 8325–4 (pCN*sesC*); iv) 8325–4 (pCN*sesC*) pre-incubated with pre-immune IgGs (80μg/ml); v) 8325–4 (pCN*sesC*) pre-incubated with αSesC-IgGs (80μg/ml). Since we expected similar impact for pre-immune IgG and αSesC-IgGs on 8325–4, we considered just αSesC-IgGs. Each inoculum was administered to each mouse through the lumen of the implanted catheter.

### Statistical analysis

Analyses of data were pooled from at least two independent experiments, and were performed using GraphPad prism 6 software. The data from *in vitro* and *in vivo* experiments involving wild-type, mutant and transformed strains were subjected to a one-way analysis of variance (1-way ANOVA) to find significant differences. A *P* value of <0.05 was considered a significant difference. Data for the transformants carrying mock plasmids are not always shown as they did not show any significant difference with wild-type conditions (data are available upon request).

## Results

### Heterologous expression of *sesC* in *S*. *aureus* switches the biofilm phenotype from PIA-dependent to proteinaceous

**Fig 1:**
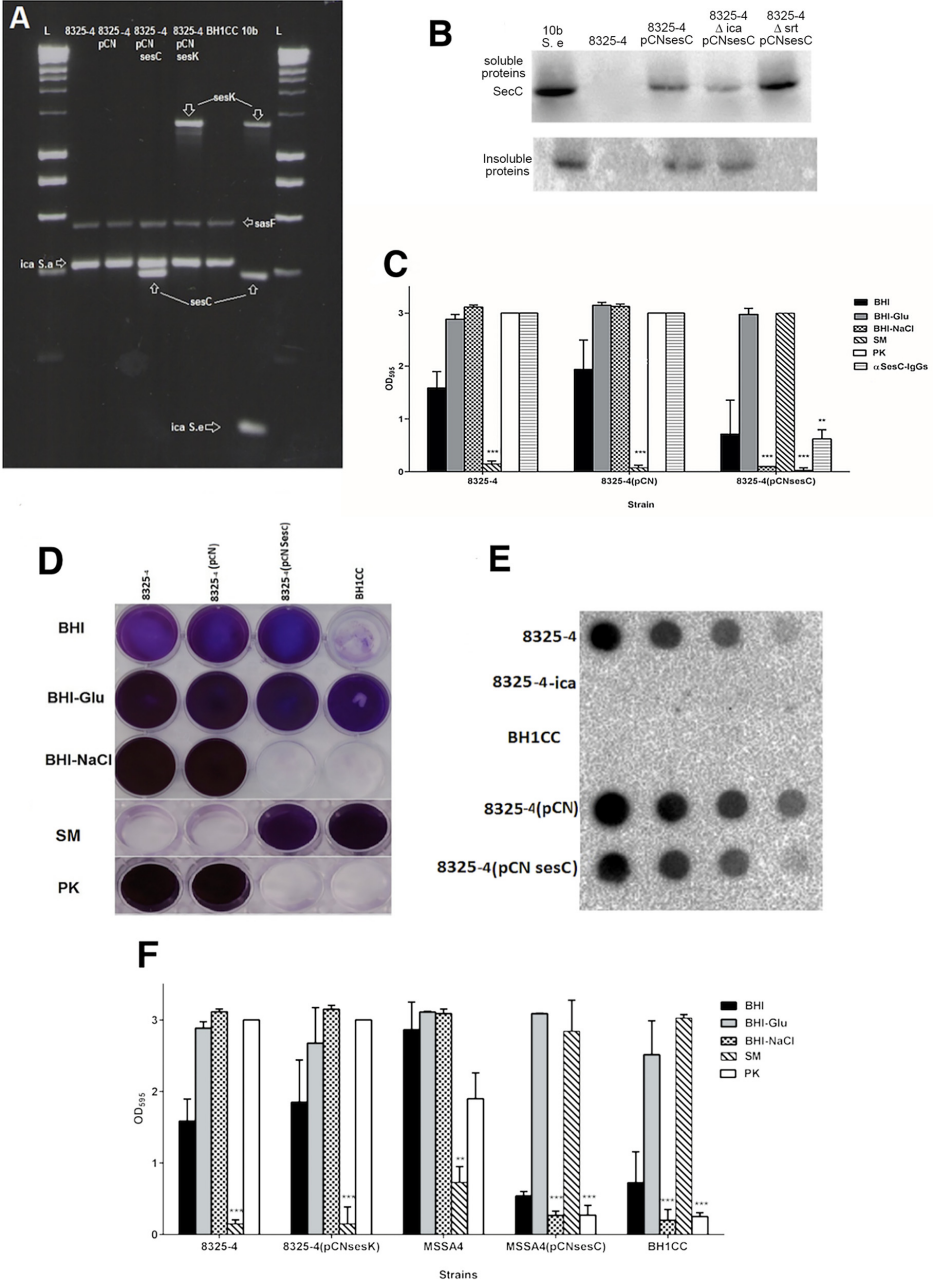
Effect of transformation of *S*. *aureus* strains with *sesC* or *sesK* on the biofilm formation. Using a semi-quantitative microtiter plate assay the level of biofilm formation in different media, the phenotype of biofilms and effect of αSesC-IgG antibodies on biofilm formation of different strains were identified. Biofilm formation in medium supplemented with NaCl or the effect of dispersal agents were used to discriminate the phenotype of biofilms. **(A**) Expression of *sesC*, *sesK*, *sasF* and *icaA* in cDNA of transformed strains was evaluated using the gel-based reverse transcription-PCR assay. **(B**) Detection of SesC production in *S*. *epidermidis* 10b, 8325–4, 8325–4 (pCN*sesC*) and 8325–4 *ica*::*tet* (pCNsesC) 8325–4 *srt*::*tet* (pCNsesC)) in soluble and insoluble fractions via Western blot assay. (**C**) The effect of *S*. *aureus* 8325–4 transformation with *sesC*. Introduction of a plasmid carrying *sesC* but not the mock plasmid changed the biofilm formation of transformants in BHI-NaCl and also the effects of PK and SM were opposite. (**D**) Microtiter plate assay demonstrating the effect of the inducers of biofilm formation (glucose and NaCl) and dispersal agents on biofilm formation of strains **(E)** quantification of PIA production using PIA non-specific immunoblot assay for biofilm in BHI-Glu. (**F**) Effect of transformation with *sesK* on the biofilm formation of strain 8325–4 and also the effect of *sesC* on the biofilm formation of strain MSSA4 in comparison with MRSA strain BH1CC. (SM: sodium metaperiodate, PK: proteinase K; error bars mean standard deviation)

The *S*. *epidermidis sesC* gene was cloned into a pCN plasmid resulting in pCN*sesC*. The recombinant plasmid was introduced into the laboratory *S*. *aureus* strain 8325–4, which makes a PIA-type biofilm, and into the hospital-associated MRSA strain BH1CC, which makes a eDNA and proteinaceous biofilm (AtlE/FnBP-dependent) [14, 19, 29]. The expression of *sesC* and the presence of the corresponding protein were confirmed by gel-based RT-PCR assay and Western blot assay (Fig 1A and 1B). Western blot confirmed the expression of SesC in the transformant strains. Heterologous expression of *sesC* had no effect on the BH1CC biofilm phenotype (data not shown), but inhibited biofilm formation by 8325–4 transformants cultivated in BHI-NaCl (Fig 1C and 1D). Furthermore, 8325–4 (pCN*sesC*) biofilms grown in BHI-glucose were dispersed with proteinase K (PK) but not sodium metaperiodate (SM), which did disperse wild-type 8325–4 biofilms (Fig 1C and 1D). This is consistent with a biofilm phenotypic switch from PNAG- to protein-mediated in these transformants. Nevertheless, quantification of PIA showed no changes in the rate of PIA production in 8325–4 (pCN*sesC*) in comparison to the wild-type strain (Fig 1E).

In order to confirm the relation between SesC production and a phenotypic switch of biofilm growth, another *ica*-positive, PIA-dependent biofilm-forming *S*. *aureus* strain, the clinical isolate MSSA4, was transformed with pCN*sesC*. As observed in 8325–4, the biofilm phenotype of MSSA4 switched from PIA-dependent to proteinaceous following introduction of pCN*sesC* (Fig 1F).

Moreover, the effect of αSesC-IgGs on biofilm formation by strains 8325–4 and 8325–4 (pCN*sesC*) grown overnight in BHI-glucose was investigated. αSesC-IgGs had no effect on 8325–4 or 8325–4 carrying the empty plasmid, but inhibited biofilm formation by 8325–4 (pCN*sesC*) up to 80% (Fig 1C and 1D). Treatment with DNaseI did not have any significant impact on the biofilms (data not shown).

**Fig 2:**
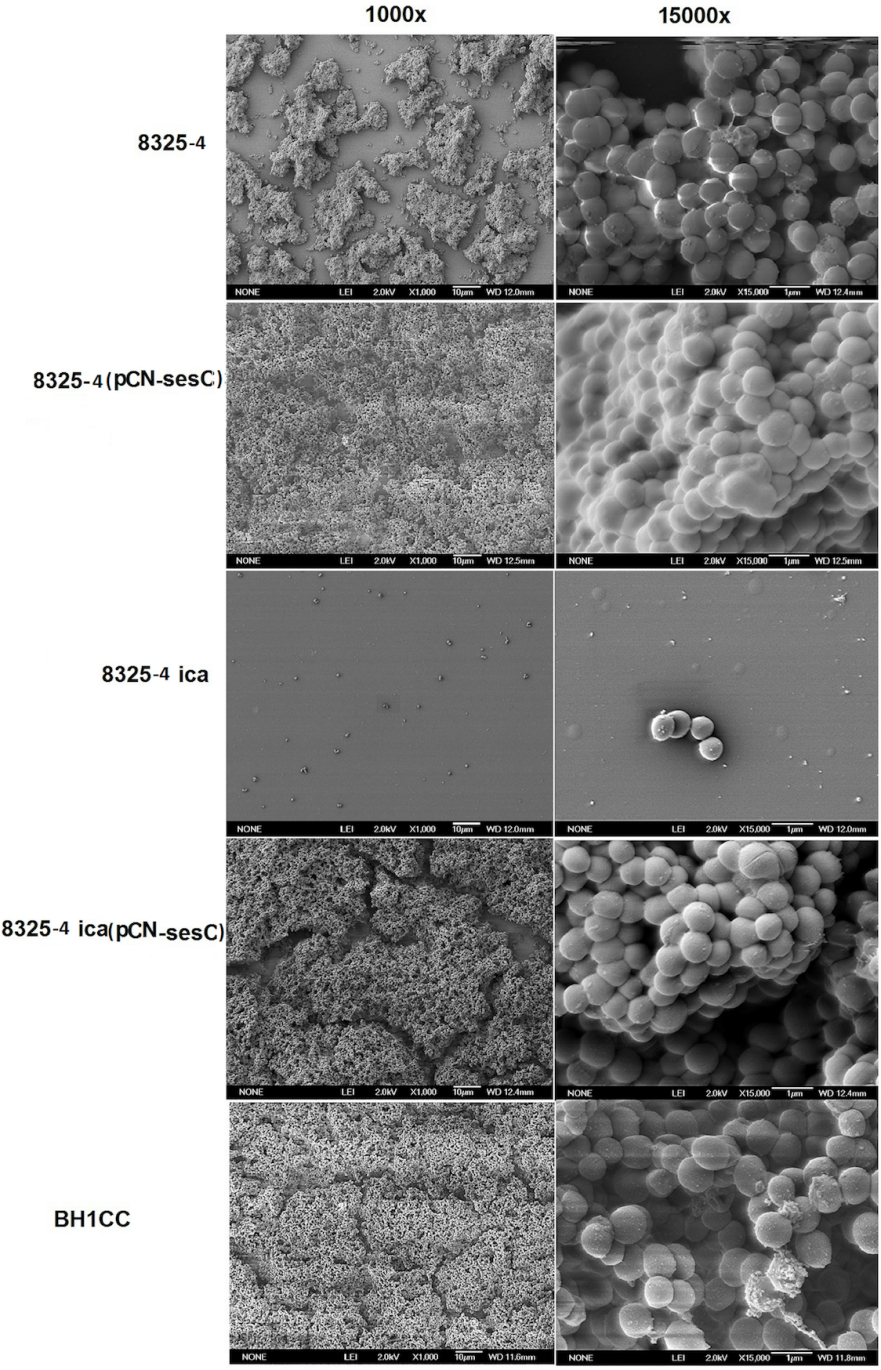
SEM images of biofilms formed by 8325–4, its *sesC*-expressing transformants and BH1CC as controls. Biofilm growth on glass disks was allowed during overnight incubation at 37°C in BHI supplemented with 1% glucose. The next day, samples were fixed and sputter coated with platinum. The images show bacteria attached on the surface of disks at 1000x, 15000x magnification.

Scanning electron microscopy (SEM) images from 8325–4, 8325–4 with mock plasmid, 8325–4 (pCN*sesC*) and BH1CC biofilms, respectively, showed morphological differences (Fig 2). In BHI-glucose, strain 8325–4 formed porous and less condensed biofilms whereas 8325–4 (pCNsesC) formed more condensed and smoother biofilms with a glue-like matrix as seen in BH1CC biofilms. 8325–4 *ica*::*tet* did not form biofilm whereas 8325–4 ica::tet (pCNsesC) presented as a biofilm forming strain (Fig 2). To evaluate whether the phenomenon of biofilm phenotypic switching is due to the specific function of SesC or to high-level constitutive production of any LPxTG surface protein, the *sesC* gene in pCN*sesC* was replaced with *sesK* that encodes another LPxTG protein in *S*. *epidermidis*. Unlike s*esC*, which is present in all *S*. *epidermidis* strains, *sesK* is only present in circa 10% of *S*. *epidermidis* isolates [28]. Additionally, it was previously shown that anti-SesC antibodies could reduce *S*. *epidermidis* biofilm formation, whereas anti-SesK antibodies had no effect [28]. Transformation with pCN*sesK* had no impact on the biofilm phenotype of 8325–4 (Fig 1F), showing that the effect of heterologous expression of *sesC* in PIA-producing *S*. *aureus* may be specific.

### Expression of *sesC* promotes biofilm production by an *ica* mutant of 8325–4

**Fig 3:**
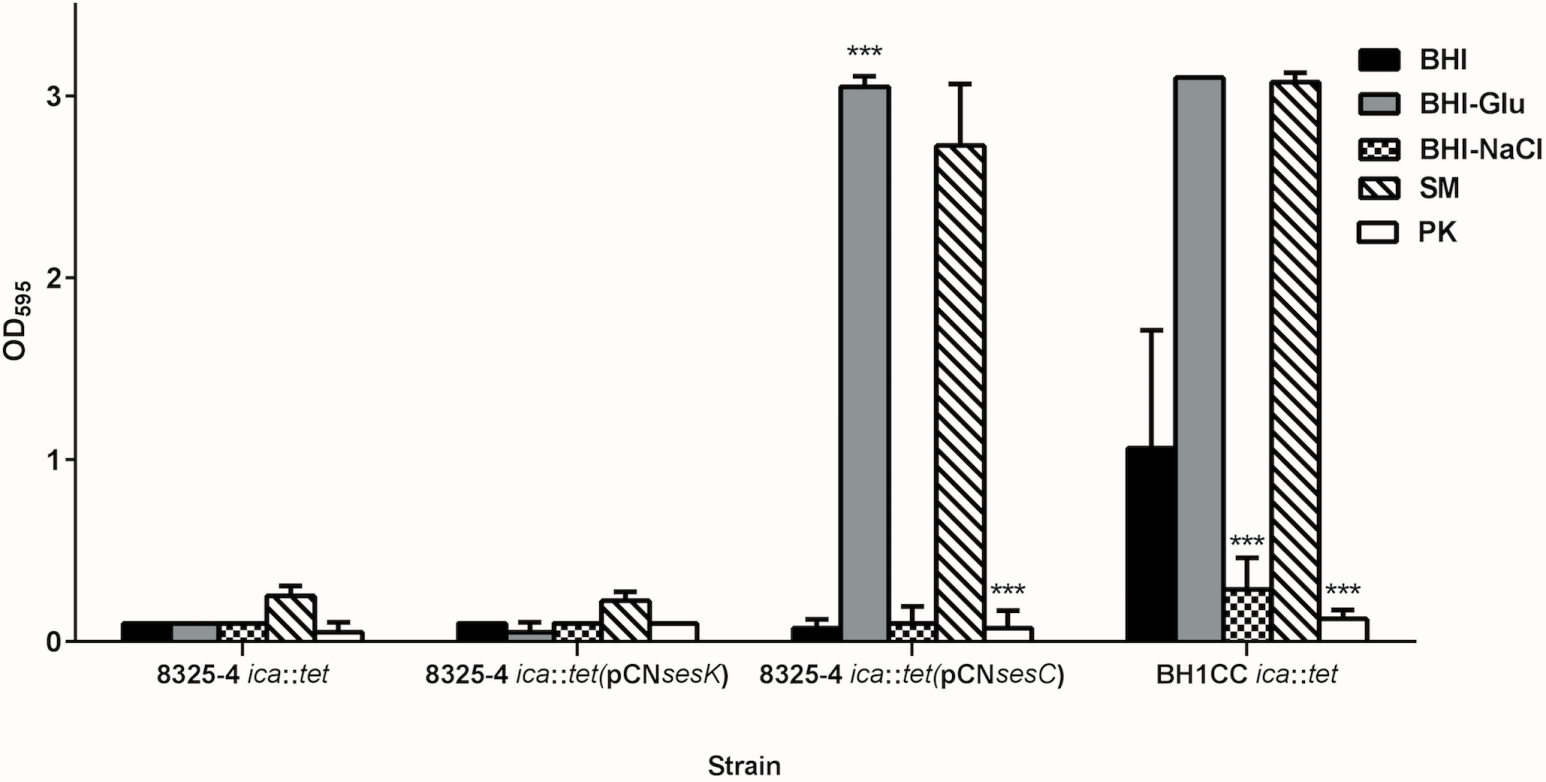
Effect of transformation with *sesC* and *sesK* on biofilm formation by non-biofilm-forming 8325 *ica*::*tet* mutant. Transformation of this non-biofilm-forming mutant with *sesC* restored its biofilm formation and converted it to protein-mediated biofilm-forming strain that cannot form biofilm in the presence of NaCl and is dispersed by PK but not SM. Transformation with *sesK* had no effect. Mutation of the *ica* in PIA-independent biofilm-forming BH1CC strain had no effect on its biofilm formation. (SM: sodium metaperiodate, PK: proteinase K; error bars mean standard deviation)

Previously published data revealed that deletion of the *ica* operon encoding PIA biosynthesis impaired PIA-dependent biofilm production by 8325–4 but had no impact on the biofilm formation by MRSA strain BH1CC which expresses an AtlE/FnBP-mediated biofilm phenotype [14]. Transformation of 8325–4 *ica*::*tet* with *sesC*—in contrast to *sesK*—restored the biofilm formation to approximately wild-type levels in BHI-glucose (Fig 3). Furthermore, SEM analysis showed that the morphology of 8325–4 *ica*::*tet* (pCN*sesC*) biofilms was similar to 8325–4 (pCN*sesC*) biofilm (data not shown). Interestingly, when grown in medium supplemented with NaCl, which induces a PIA-type biofilm, 8325–4 *ica*::*tet* (pCN*sesC*) was unable to produce biofilm while in BHI-glucose biofilm growth of 8325–4 *ica*::*tet* (pCN*sesC*) occurred. In addition, these biofilms were dispersed by PK, indicating a proteinaceous biofilm phenotype for this transformed mutant (Fig 3).

### Surface expression of SesC is involved in biofilm formation

**Fig 4:**
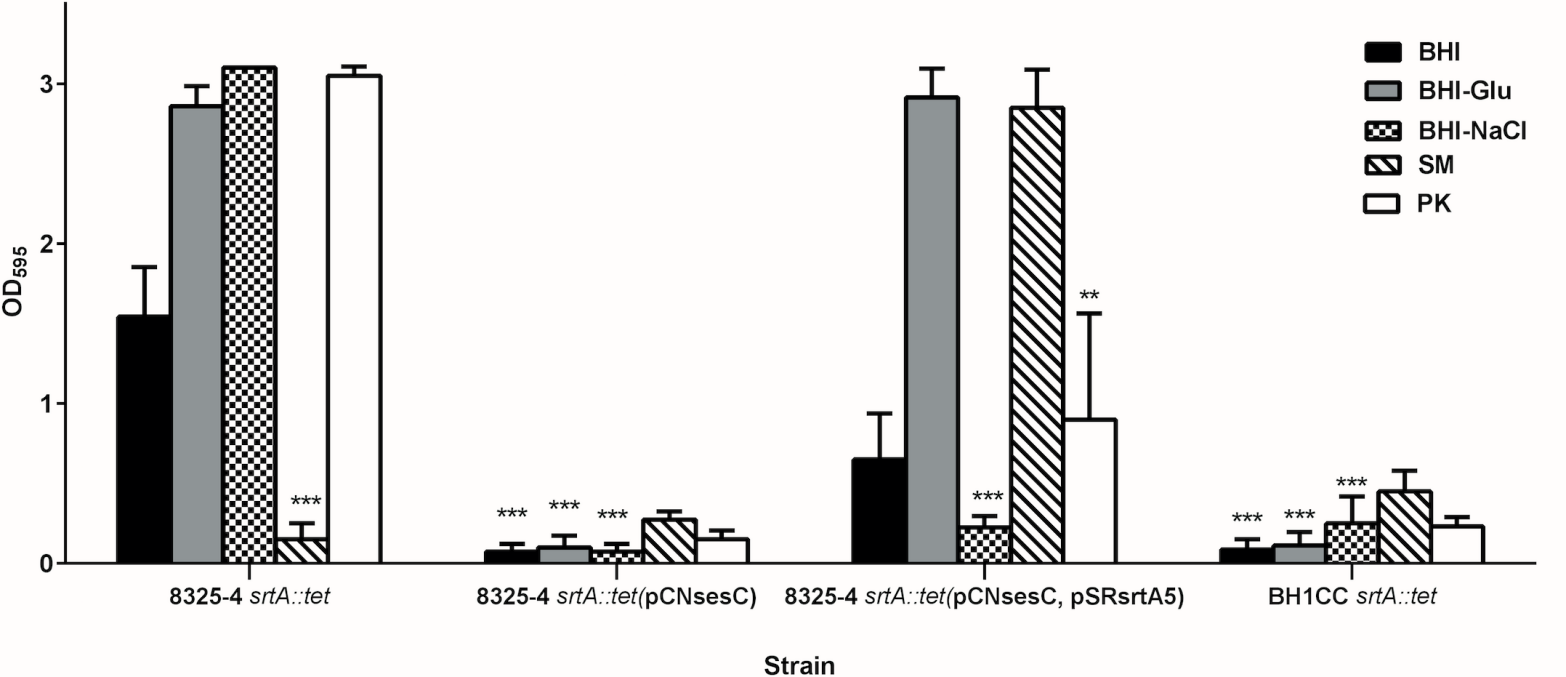
Effect of transformation with *sesC* on biofilm formation by the biofilm-forming isogenic *srtA* mutants of 8325–4. Effect of *srtA* mutation on biofilm formation of PIA-dependent biofilm-forming strain 8325–4, (Atl/FnBP)-mediated biofilm-forming strain BH1CC, the *sesC*-tranformed 8325–4 *srtA*::*tet* strain and complementation of 8325–4 *srtA*::*tet* (pCN*sesC*) with *srtA*, was evaluated using the quantitative microtiter plate assay. (SM: sodium metaperiodate, PK: proteinase K; error bars mean standard deviation)

LPxTG proteins are known to be anchored to the bacterial peptidoglycan by sortases [37, 38, 39]. Deletion of *srtA* inhibits LPxTG protein-dependent biofilm formation. In 2008, O'Neill *et al*. reported that deletion of *srtA* in BH1CC impairs its biofilm forming activity, while biofilm formation of 8325–4 *srtA*::*tet* was not affected [26]. Introduction of pCN*sesC* in the latter strain, however, completely impaired biofilm production, further indicating the dominant role of SesC over PIA-type biofilm production (Fig 4). Unlike 8325–4, in the absence of sortase the 8325–4 *srtA*::*tet* (pCN*sesC*) strain was unable to form biofilm. The presence of SesC in the soluble and insoluble proteins fractions of the transformant strain was investigated and showed that in the 8325–4 *srtA*::*tet* (pCN*sesC*) SesC is only expressed in the soluble fraction (Fig 1B). This strongly suggests that presence of *srt* is necessary for sorting SesC to the cell wall.

Complementation of 8325–4 *srtA*:: *tet* (pCN*sesC*) with the plasmid pSR*srtA*5 carrying the *S*. *aureus srtA* gene [26] restored biofilm formation (Fig 4). The biofilm of this complemented strain was only induced in BHI-glucose 1%, not in BHI-NaCl, and dispersed only with Proteinase K and not with sodium metaperiodate.

### Heterologous expression of *sesC* increases colonization of polyurethane intravenous catheters *in vitro* and *in vivo*

**Fig 5:**
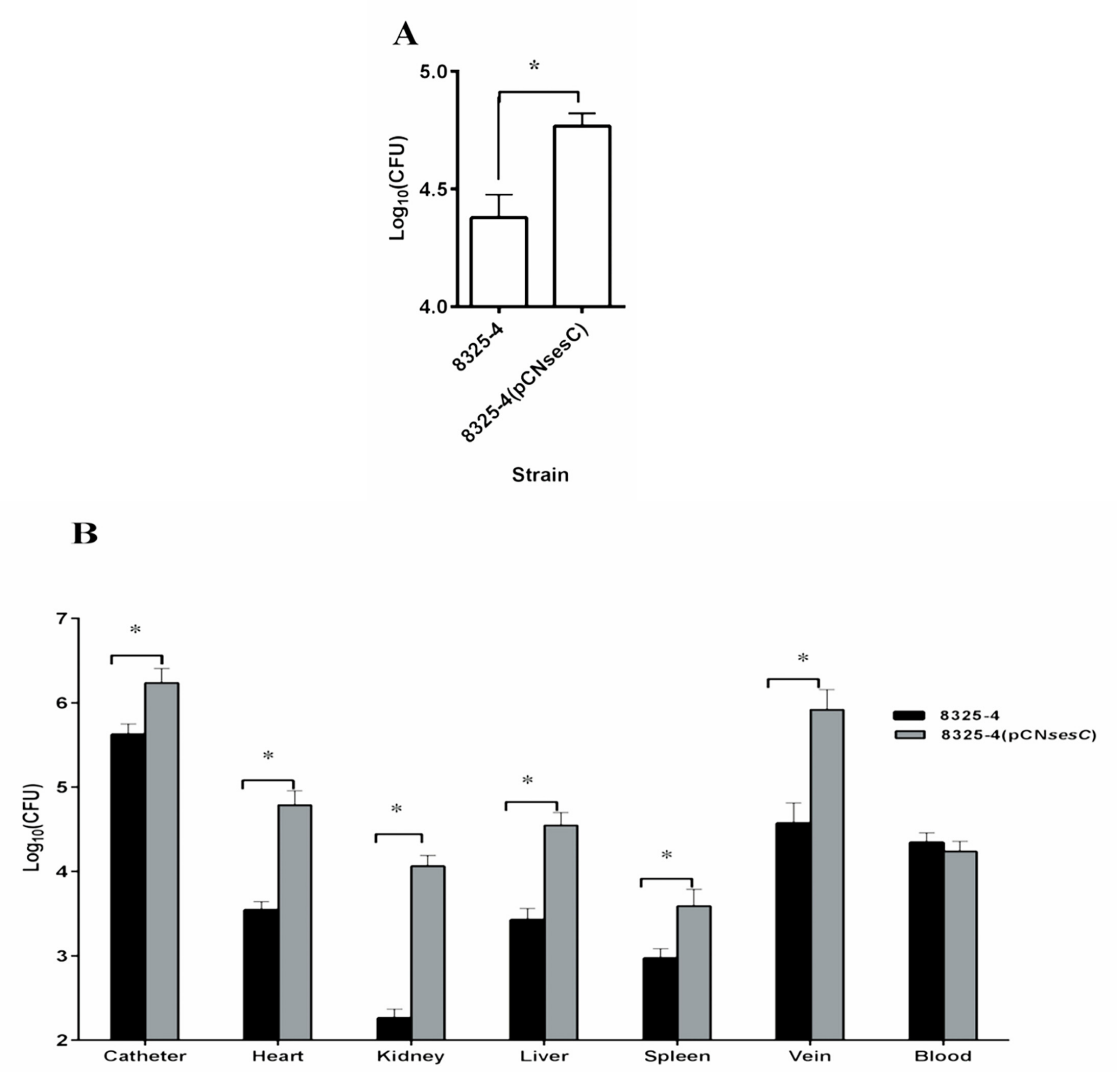
Effect of transformation with *sesC* on *in vitro* and *in vivo* catheter colonization and *in vivo* organ colonization. (**A**) Seven mm catheter fragments were inoculated with 8325–4 or its *sesC*-expressing transformant. After 2 h incubation at 37°C, catheters were rinsed and adherent bacteria were detached by sonication and the numbers of bacteria recovered from each catheter fragments were quantified by quantitative cultures. (**B**) *In vivo*, catheterized animals were inoculated through the catheter lumen with 8325–4 strain or its 8325–4 (pCN*sesC*) transformant. At day 5 post-infection, the numbers of bacteria attached to the catheters or recovered from organs were quantified by quantitative cultures. The *sesC*-expressing transformant 8325–4, (pCN*sesC*) has a higher rate of colonization of different organs up to 100-fold compare to its parental strain. *: *P*<0.05

Transformation of *S*. *aureus* 8325–4 with *sesC* significantly increased its attachment to polyurethane intravenous catheters *in vitro* (*P*<0.01; 1-way ANOVA) (Fig 5A). Using a jugular vein catheterized (JVC) mouse model, the number of bacteria recovered from the catheter implanted in animals and afterwards infected with 8325–4 (pCN*sesC*) was also significantly higher than after administration of 8325–4 (*P*<0.05; 1-way ANOVA) (Fig 5B). Interestingly, 8325–4 (pCN*sesC*) did not only show an increased catheter colonization ability compared to the non-transformed strain, but also the overall infection rate was raised. The number of 8325–4 *versus* 8325–4 (pCN*sesC*) cells in blood were similar, but the number of 8325–4 (pCN*sesC*) cells recovered from organs such as spleen, liver, heart, vein and kidney were significantly increased compared to 8325–4 (10- to 100-fold, *P*<0.05; 1-way ANOVA) (Fig 5B). These results indicate that SesC is a colonization factor that may promote *S*. *epidermidis* catheter colonization, which in turn is the first step in biofilm formation and the establishment of a chronic infection.

### Antibodies against SesC have therapeutic benefit in an 8325–4 (pCN*sesC*)-induced catheter-related infection

**Fig 6:**
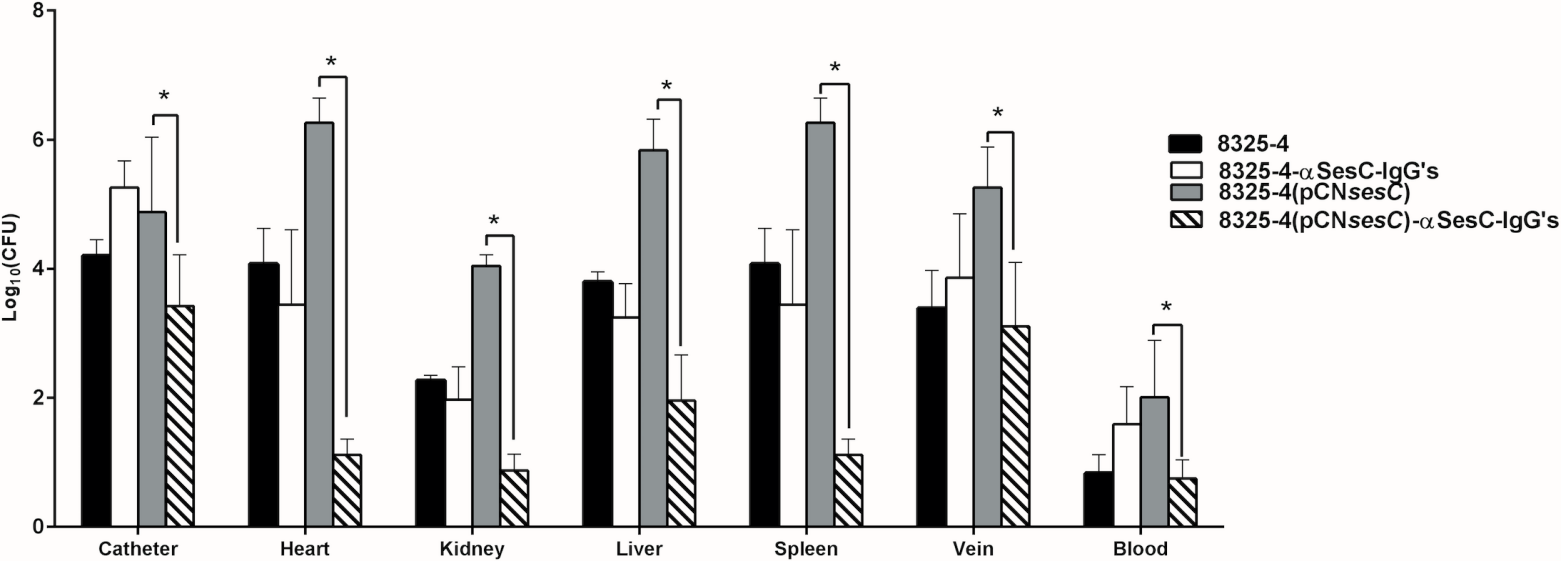
Effect of αSesC-IgGs on catheter colonization and infection rate by 8325–4 and its *sesC*-expressing transformant. Catheterized animals were inoculated with bacteria pre-incubated with αSesC-IgGs or pre-immune IgGs for 2 h at 4°C. The next day, animals were sacrificed and the bacteria were recovered from catheter and organs and quantified by quantitative cultures. Pre-incubation with αSesC-IgGs significantly reduced the rate of catheter and organ colonization by 8325–4 (pCN*sesC*) from 10 to 10000-fold. *p<0.05

The rate of catheter and organ colonization significantly decreased (100–100.000 fold; *P*<0.01–0.001; 1-way ANOVA) in the JVC mouse model group inoculated with 8325–4 (pCN*sesC*), which were pre-incubated with αSesC-IgGs *versus* untreated 8325–4 (pCN*sesC*) (Fig 6). Pre-immune IgGs had no significant effect on the catheter and organ colonization by 8325–4 or its *sesC*-expressing transformant (data not shown).

## Discussion

Surface proteins have been shown to play important roles in *S*. *epidermidis* and *S*. *aureus* biofilm formation, especially by MRSE and MRSA in device-related infections [10, 14, 18, 40]. We previously reported that rabbit polyclonal antibodies directed against the extracellular domain of *S*. *epidermidis* LPXTG surface protein SesC (or αSesC-IgGs) can significantly inhibit *S*. *epidermidis* biofilm formation *in vitro* and *in vivo* in a rat model of subcutaneous catheter-related infection (CRI) as well as in a mouse model of jugular vein CRI [23, 28]. It has also been demonstrated that active immunization with the recombinantly produced extracellular domain of SesC decreased *S*. *epidermidis* biofilm formation in a rat model of subcutaneous CRI [28]. Data obtained in this study are consistent with previous observations and demonstrate that SesC plays an important role in biofilm formation even in another genetic background.

We have tried, but were unable to knockout *sesC*. It may be that knockout of *sesC* is associated with a lethal phenotype in these bacteria. On the other hand, using antisense RNAs to knock down *sesC* in *S*. *epidermidis* and subsequently applying gel-based reverse transcription-PCR (RT-PCR) assay and Western blot analysis, we could not see any changes in *sesC* expression in the transformed strain compared with the parental strain.

PCR screening to determine the incidence of *sesC* in 300 clinical isolates of *Staphylococcus* spp. including 175 *S*. *epidermidis*, 33 isolates of methicillin-resistant *S*. *aureus* (MRSA), 50 isolates of methicillin-sensitive *S*. *aureus* (MSSA) and 42 strains belonging to various non-*epidermidis* CoNS species, revealed that *sesC* is present in all *S*. *epidermidis* strains, but not in other staphylococci, indicating that it is potentially a conserved, *S*. *epidermidis-*specific gene.

Despite the lack of *S*. *epidermidis sesC* mutants, the unraveling of the function of SesC in biofilm formation was attempted by introduction of *sesC* into *S*. *aureus* strains that formed different types of biofilm (PIA-dependent, PIA-independent or non-biofilm-forming). Using gel-based RT-PCR assay and Western blot analysis, we confirmed *sesC* expression and the production of the corresponding protein in these transformed strains. Our findings revealed that transformation with *sesC* had no impact on the strains, which display proteinaceous biofilm phenotypes, while it caused a switch from PIA-dependent biofilm-formation to a proteinaceous-type biofilm in MSSA strains 8325–4 and MSSA4. As this biofilm phenotype switching was not associated with transformation of 8325–4 with *sesK*, it can be concluded that the switch is not necessarily associated with high expression level of any LPxTG surface protein or with the genetic background of the transformed strain, but is specifically caused by SesC. Results obtained also show that presence of SesC is dominant over the presence of PIA and impairs the role of PIA in cell-cell interaction.

To further confirm the direct involvement of SesC in biofilm formation, we transformed the non-biofilm-forming, isogenic *ica* mutant of *S*. *aureus* strain 8325–4 (being 8325–4 *ica*::*tet*) with *sesC*. Transformation with *sesC* converted this non-biofilm-forming mutant to a proteinaceous biofilm-forming strain similar to its parental strain transformed with *sesC*. These data suggest a direct role for SesC in biofilm formation, because even in the absence of PIA we have the same effect of SesC on biofilm formation.

To answer the question whether SesC affects PIA biofilm formation in mutated *srtA* strain, *S*. *aureus* 8325–4 *srtA*::*tet* was transformed with *sesC*. Deletion of *srtA* has no impact on biofilm formation of PIA-dependent biofilm-forming strain 8325–4, but impairs the normal display of LPxTG surface proteins, including SesC. After the introduction of *sesC*, we observed that this biofilm-forming strain converted to a non-biofilm-forming strain. To confirm the localization of SesC in 8325–4 *srtA*::*tet* (pCNsesC) strain, we checked the presence of SesC in the soluble and insoluble protein fraction. Since SesC is a cell wall-anchored LPXTG protein, basically we expected to find it in the insoluble fraction. However, SesC was observed only in the soluble part. These data suggest that the presence of sortase is necessary to sort SesC to the cell wall [41].

Complementation of the non-biofilm-forming *sesC*-expressing mutant with *srtA* by means of transformation with pSRsrtA5 converted it to a proteinaceous biofilm-forming strain. These data confirm that transformation with *sesC* is sufficient to switch the mechanism of biofilm formation to a proteinaceous type biofilm on condition that SesC is sorted to its place on the surface, attached to the peptidoglycan layer. Our data show that SesC expression does not have any negative effect on PIA production. There is some evidence that may explain this phenomenon. Previous groups reported that the generation of PIA is not sufficient to form biofilm [42]. Vergara-Irigaray *et al*. showed that the clinical MRSA strain 132 is able to alternate between a proteinaceous and a polysaccharidic biofilm matrix, depending on environmental conditions, and strain S115 generates PIA but is a non-biofilm-forming strain. This might be because of the existence of a defect in the export of PIA by IcaC or IcaB [42]. Similarly to the effect of FnAB on biofilm phenotype in *S*. *aureus* strain S132 and BH1CC, there is the possibility that here, the extracellular location of SesC changes the architecture of the cell wall to the extent that PIA can no longer link the cells. This hypothesis is consistent with the observed effect of biofilm dispersal agents on *sesC-* and *ica*-positive strains. The observation that high level expression of another surface protein (SesK) did not have a similar effect may suggest some form of interaction between SesC and PIA that is absent for SesK. Different expression levels of SesC and SesK in spite of using the same expression vectors may offer another explanation.

Expression of SasG, a surface protein in *S*. *aureus*, can similarly as SesC switch the biofilm of PIA-dependent strains SH1000 and 8325–4 to protein-mediated biofilm [39] SasG like SesC is a fibrinogen-binding protein, and SasG masked binding to fibrinogen mediated by both ClfB and the FnBPs. Biofilm formation by SasG is also likely to be protease dependent, because the broad spectrum protease inhibitor a2-macroglobulin inhibited the biofilm formation process of the strain SH1000 transformed with *sasG* [39]. By looking at the protein sequences alignment of SesC and SasG, we realized they have 26% identity.

Similar to the presented observations, a recent report illustrated the impact of introducing the methicillin resistant gene *mecA* into the PNAG-producing MSSA strain 8325–4 [19]. This generated a heterogeneously oxacillin resistant (HeR) strain, from which a homogeneous, high-level resistant (HoR) derivative was isolated following exposure to oxacillin. Transcription of *icaADBC* and production of PNAG were impaired in the 8325–4 HoR derivative, which instead produced a proteinaceous biofilm that was significantly inhibited by antibodies against the *mecA*-encoded penicillin binding protein 2a (PBP2a). HoR derivatives of 8325–4 *icaADBC*::*tet*, 8325–4 *fnbAB*::*tet*, 8325–4 *atl*::*cat* and 8325–4 *srtA*::*tet* exhibited a similar biofilm phenotype as 8325–4 HoR [19, 43].

SEM images confirm the presence of a morphologically different biofilm for 8325–4 in comparison to its transformant harboring pCN*sesC*. These findings are also supported by the effect of αSesC-IgGs on established biofilms of SesC-producing transformants and also *in vitro* catheter colonization data that suggest a role for SesC in attachment to the catheter surfaces.

The obtained *in vivo* data are consistent with our previous findings in suggesting an important role for SesC in infection. Transformation with *sesC* increased the organ infection rate up to 100-fold. This can be partially explained by the fact that fibrinogen is one of the components of the extracellular matrix [44]. Our previous report showed that transformation of *S*. *aureus* strain RN4220 with *sesC* increased the fibrinogen-binding ability of transformants, suggesting SesC as a potential Fg-binding MSCRAMM [23]. But, our experiments also show enhanced adherence of the transformant strains to an uncoated catheter in vitro, suggesting that the adherence effects are not entirely or solely mediated by binding to host factors.

Reduction of *in vivo* catheter and organ colonization by SesC-producing *S*. *aureus* strains in the presence of αSesC-IgGs indicate the specificity of the antibody, surface expression of SesC, and involvement of SesC in catheter and organ colonization.

Although we have to be cautious in extrapolating conclusions based on data obtained in *S*. *aureus* to *S*. *epidermidis*, we conclude that SesC is a virulence factor associated with the early stages in *S*. *epidermidis* biofilm formation, such as adhesion and colonization possibly favoring chronic, persistent infections on indwelling biomaterials. The biofilm formation versatility and flexibility of *S*. *epidermidis* may be due in part to the presence of SesC and similar factors that help *S*. *epidermidis* to adapt to changing environmental conditions.

In future studies, SesC can be considered as a valuable vaccine target against *S*. *epidermidis* infections.
